# Acknowledgement to Reviewers of *Viruses* in 2018

**DOI:** 10.3390/v11010044

**Published:** 2019-01-09

**Authors:** Viruses Editorial Office

**Affiliations:** MDPI, St. Alban-Anlage 66, 4052 Basel, Switzerland

Rigorous peer-review is the corner-stone of high-quality academic publishing. The editorial team greatly appreciates the reviewers who contributed their knowledge and expertise to the journal’s editorial process over the past 12 months. In 2018, a total of 721 papers were published in the journal, with a median time to first decision of 16 days and a median time to publication of 37 days. The editors would like to express their sincere gratitude to the following reviewers for their cooperation and dedication in 2018:
Abdelwhab, El-SayedLindenbach, BrettAbdul-Careem, Mohamed FaizalLing, ChenAbedon, StepehLinia, MaxineAbergel, ChantalLinial, Maxine L.Accardi, RositaLiu, SijunAccotto, GianLiu, WeiAdams, Sandra D.Liu, XiaoyingAdler, HeikoLiu, XinfengAdriaenssens, EvelienLiu, YueAdriaenssens, Evelien M.Lo, Shih-YenAffabris, ElisabettaLo, SzechengAgarkova, Irina VLobocka, MalgorzataAghemo, Alessio MicheleŁobocka, MałgorzataAgudelo-Romero, PatriciaLodge, RobertAgui, TakashiLodmell, J. StephenAhi, YadvinderLong, Heather M.Airenne, KariLopalco, LuciaAkula, ShawLopez-Ferber, MiguelAlbertini, MariangelaLópez-Moya, Juan JoséAlbrecht, RandyLou, FangfeiAlcaine, SamLoughran, GaryAlcorn, JohnLozach, Pierre-YvesAldskogius, HåkanLu, HuasongAlejo, AlíLucifora, JulieAlfson, KendraLuetgehetmann, MarcAliota, MatthewLukac, DavidAlizon, SamuelLukashevich, IgorAlmazan Toral, FernandoLukashevich, Igor S.Almendral, JoséLukhovitskaya, NinaAlmodovar, SharilynLundstrom, KennethAlteri, ClaudiaLuo, DahaiAltomonte, JenniferLusic, MarinaAlvisi, GualtieroLuthra, PriyaAmarasinghe, GayaLy, HinhAmarilla, Alberto A.Ma, ZexuAn, DongJunMacDonald, MargaretAnany, HanyMaciejewska, BarbaraAnastasina, MariaMackenzie, JasonAndo, SugihiroMackow, Erich R.Andonov, AntonMadan, VanesaAndres, GermanMaggirwar, Sanjay BAndrophy, Elliot J.Mahony, Timothy JohnAngeletti, PeterMainou, BernardoAnnunziata, GiuseppeMalik, DanishAnsaldi, MireilleManicassamy, BalajiAparicio, FredericMantis, NicholasApperson, CharlesManzoor, RashidAquaro, StefanoMarais, ArmelleArbuckle, JesseMarc, DanielArcangeletti, Maria-CristinaMarcello, AlessandroAriën, Kevin K.Marchant, DavidAriyasu, DaisukeMaree, Hano J.Arkhipova, KseniaMargaria, PaoloArmon, RobertMaria Vaira, AnnaArnold, MichelleMarr, NicoArruda, Bailey L.Marsh, MarkArtyszak, ArkadiuszMarthaler, Douglas G.Ascencio-Ibanez, TrinoMartin, AnnetteAsfor, Amin S.Martín, VerónicaAtamian, HagopMartina, ByronAuguste, Albert J.Martín-Acebes, MiguelAuray, GaelMartinelli, ElenaAuvray, FrédéricMartínez, GermánAvadhanula, VasanthiMartínez-Avilés, MartaAvrani, SaritMartínez-García, ManuelAwasthi, SitaMartinez-Gil, LuisAzab, WalidMartínez-Gómez, PedroAzeredo, JoanaMartínez-Salas, EncarnaciónBabiuk, ShawnMartins, NelsonBachmann, MartinMarzano, Shin-YiBag, SudeepMarzocco, StefaniaBaiocchi, Robert A.Mascia, TizianaBaird, NicholasMasłyk, MaciejBakonyi, TamásMasuda, HisakoBakre, Abhijeet A.Matczuk, Anna KarolinaBaliraine, FrederickMatosiuk, DariuszBalistreri, GiuseppeMatoušek, JaroslavBalkema-Buschmann, AnneMatsukura, KeiichiroBamford, ConnorMatsushita, YosukeBańbura, Marcin W.Matsuzaki, ShigenobuBandea, ClaudiuMatsuzaki, YokoBarbirz, StefanieMatthews, BenBarbu, MagdaMatthijnssens, JelleBarik, SailenMayer, JensBartee, EricMcCullagh, Martin J.Bartual, Sergio G.McDaniel, Lauren D.Basso, Thiago OlittaMcGregor, AlistairBatchu, RameshMcInerney, GeraldBates, John T.McSharry, Brian PatrickBatuman, OzgurMcVey, David ScottBeard, MichaelMeckes, DavidBeatty, JuliaMedin, Carey L.Beatty, P. RobertMedveczky, PeterBecker, Stefanie ChristineMeeus, IvanBeckham, J. DavidMelero, José AntonioBeemon, KarenMelo, LuisBego, Mariana G.Mena, IgnacioBehrens, Sven-ErikMenéndez-Arias, LuisBej, AsimMeng-Ji, LuBelaganahalli, ManjunathaMenon, VipinBellés, José MaríaMergia, AyalewBelouzard, SandrineMerino, José JoaquínBelov, GeorgeMerits, AndresBente, DennisMerola, MarcelloBergeron, EricMertens, ThomasBerlioz-Torrent, ClarisseMeseda, ClementBernacchi, SerenaMeškys, RolandasBernard, KristenMesquita, JoãoBerthoux, LionelMessaoudi, IlhemBertke, AndreaMetz, Stefan W.Bertoni, GiuseppeMeulia, TeaBertran, KateriMeyer, FlorenciaBerzal-Herranz, AlfredoMeyers, CraigBevins, CharlesMiao, HongyuBhagwat, AshokMiesen, PascalBhattacharjee, ApurbaMilavetz, BarryBidgood, SusannaMillard, AndrewBiegalke, BonitaMiller, MattBielefeldt-Ohmann, HelleMiller, Sara EBierle, CraigMiller, W. AllenBikard, DavidMinafra, AngelantonioBinder, MarcoMiozzi, LauraBinley, KatieMirazimi, AliBirlea, MariusMire, ChadBistolas, Kalia S. I.Miroshnikov, KonstantinBlack, LindsayMiyazawa, MasaakiBlair, CarolMlera, LuwanikaBlasdell, KimMocarski, EdwardBlazquez, Ana-BelenModhiran, NaphakBloom, MarshallMöhl, BrittaBloom, Marshall E.Moineau, SylvainBlutt, Sarah ElizabethMoons, ChristelBocharov, GennadyMoore, Matthew D.Bock, ThomasMora, DiegoBodily, JasonMoratorio, GonzaloBoehme, KarlMordecai, GideonBohn, ErwinMoreland, NikkiBollinger, TrentMoreno, AnaBoonstra, AndréMorgan, Iain M.Bopegamage, ShubhadaMori, HajimeBorca, ManuelMoriones, EnriqueBose, SayantanMoriyama, HiromitsuBosse, Jens B.Morona, RenatoBotella, LeticiaMorozov, IgorBotten, JasonMorrison, Lynda A.Bourgeois-Daigneault, Marie-ClaudeMorrison, Thomas E. "Tem"Bowen, RichardMoser, Lindsey A.Bradfute, StevenMossel, EricBradley, KonradMotwani, TinaBradrick, Shelton S.Moutelikova, RomanaBrandt, CurtisMühlberger, ElkeBrasel, TrevorMukhopadhyay, TuliBratbak, GunnarMunger, JoshuaBratkovic, TomazMunoz-Fontela, CesarBrault, AaronMurakami, YoshikiBrault, VéroniqueMurata, TakayukiBravo, IgnacioMuriaux, DelphineBrennan, GregMurooka, ThomasBrenner, Bluma G.Murphy Schafer, AshleighBreyton, CecileMurphy, BrianBriant, LaurenceMurphy, EainBrien, James D.Murray Stewart, TracyBrindley, MelindaMuthaiyan, ArunachalamBrinkmann, MelanieMutinelli, FrancoBrinsmade, ShaunMuylkens, BenoitBrinton, MargoMyoung, JinjongBrister, RodneyMyrsini Eirini, NatsopoulouBritt, WilliamNagai, MakotoBroadbent, AndrewNagano, Atsushi J.Broccolo, FrancescoNagasaki, KeizoBrochot, EtienneNagy, Peter D.Broder, ChristopherNaik, SurabhiBroker, TomNair, VenugopalBrown, EarlNakahara, Kenji S.Brown, JayNakai, HiroshiBrown, StanleyNakamoto, ShingoBrugnera, EnricoNakanishi, AkiraBrum, Jennifer R.Narasimhan, SukanyaBrun, AlejandroNarayanan, AarthiBrune, WolframNarute, PurushottamBrüssow, HaraldNasimuzzaman, MdBuck, ChrisNavarro, BeatrizBujarski, Józef J.Nawrocki, Steffan T.Bukrinsky, MichaelNejman-Faleńczyk, BożenaBullitt, EstherNelson, Daniel C.Burckhardt, ChristophNelson, Scott W.Burges, GrahamNetherton, ChrisBurkard, ChristineNeve, HorstCadwell, Ken H.Nevels, MichaelCaeiro, MariaNewcomb, Laura L.Calisher, Charles H.Nichols, DanielCalvert, Amanda E.Nicola, AnthonyCampbell, CoreyNiczyporyk, Jowita SamantaCampbell, EdwardNiikura, MasahiroCampbell, MelNiller, Hans HelmutCanuti, MartaNilsson, AndersCao, DianjunNing, ShunbinCao, PengxingNishigaki, KazuoCara, AndreaNishiguchi, MasamichiCardeti, GiusyNissimov, JozefCarlson, PaulNiu, DongyanCarr, Jillian M.Nogalez-Gonzalez, AitorCarrillo, ConsueloNorder, HeleneCarrington, ChristineNorton, PamelaCarroll, Miles W.Nüesch, JürgCasasnovas, José M.Nunberg, JackCastilletti, ConcettaNuss, Donald L.Castillo, DanielNuttall, Patricia ACen, OsmanNyström, KristinaČerný, JiříO’Connor, ChristineCeyssens, Pieter JanOglesbee, MichaelChae, ChanheeOh, Jin-WooChamakura, KarthikOhshima, KazusatoChan, Kuan RongOkamatsu, MasatoshiChang, BarbaraOkamoto, ShigefumiChang, Hui-WenOkeke, Malachy IfeanyiChang, JinhongOksanen, HannaChang, Tsung-HsienOlety, BalajiChanishvili, NinaOliver, RichardChao, MeiOlszak, TomaszChapman, Nora M.Omatsu, TsutomuChase, ChristopherOrtego, JavierChatel-Chaix, LaurentOttmann, MichèleCheeran, MaximOura, ChrisChejanovsky, NorOura, Christopher A.LChen, Hui-WenOuwendijk, Werner J. D.Chen, Mei-RuÖverby, Anna K.Chen, QiPaeshuyse, JanChen, Yu-ChihPaessler, SlobodanCheng, HanPagán, IsraelCheng, Xiao-WenPager, CaraCheshenko, NataliaPaik, Soon YoungChevalier, ChristophePaillot, RomainChiba, SotaroPajunen, MariaChiou, Shyan-SongPalmarini, MassimoChizhov, AntonPalù, GiorgioChlanda, PetrPandey, Krishan K.Cho, Nam-HyukPantůček, RomanChoi, Seung-KookPapa, AnnaChoi, Tae-JinParadkar, PrasadChoumet, ValérieParcells, MarkChowdhury, ShafiqulPardigon, NathalieChu, JustinParent, KristinChung, DonghoonParent, RomainChung, Hee-ChunParish, ColinChung, JunhoPark, EnochCillo, FabrizioPark, Hyun-WooCingolani, GinoParker, ScottCirilli, MarcoParks, Griffith D.Cirone, MaraParra, GabrielCiuffi, AngelaPasdeloup, DavidCivra, AndreaPasick, JohnClaverie, Jean-MichelPaskaleva, ElenaClem, RolliePastey, ManojClement, SophiePatel, Manish R.Cliffe, AnnaPathak, VinayCobbin, JoannaPatterson-West, JenniferCoffey, AidanPaxton, RobertCohrs, RandallPearson, MichaelColpitts, Che C.Pécheur, Eve-IsabelleColpitts, TonyaPedersen, Finn SkouComas-Garcia, MauricioPeiris, Joseph Sriyal MalikCompton, AlexPekosz, AndyConrad, NicholasPelchat, MartinConstable, FionaPellett, PhilipConzelmann, Karl-KlausPeng, ChenCoombs, KevinPereira, LeonelCoombs, Kevin MPerera, RushikaCooper, BretPérez Artés, EncarnaciónCortés, Maria PilarPerez, LaurentCortez, ValeriePerez, VicenteCosset, François-LoïcPérez-Gracia, María TeresaCosta, Ana RitaPerez-Gracia, Maria-TeresaCôté, MarcelinePerilla, Juan RobertoCotmore, SusanPerreault, Jean-PierreCoto, MairenePerreault, JonathanCouderc, ThérèsePeruzzi, FrancescaCoutts, Robert H. A.Petrzik, KarelCrawford, LindseyPeyambari, MahtabCross, RobertPfaender, StephanieCrossan, ClairePfaff, FlorianCulley, AlexanderPickett, Brett E.Cvirkaite-Krupovic, VirginijaPietilä, MaijaCysique, Lucette APijlman, GorbenD’Onofrio, AlbertoPincus, SethDabrowska, KrystynaPintel, DavidDainat, BenjaminPiret, JocelyneDalli, JesmondPisegna, Joseph R.Dalton, K. P.Pizzato, MassimoD’Andrea, Marco MariaPizzorno, Mario AndresDandri, MauraPlachter, BodoDatta, Prasun K.Plattet, PhilippeDaugelavicius, RimantasPlesko, Irena MavricDaugrois, Jean-HeinrichPlevka, PavelDavido, DavidPodgórska, KatarzynaDavis, April D.Pohlmann, StefanDavis, David A.Pöhlmann, StefanDe Andrea, MarcoPolak, Mirosław PawełDe Andrés Cara, Damián F.Polakowski, Nicholas J.De Grazia, SimonaPoli, GuidoDe La Peña, MarcosPollastro, Stefaniade la Torre, Juan CarlosPoncet, DidierDe Miranda, JoachimPope, WelkinDe Ory, FernandoPopham, Holly J.de Swart, RikPopham, Holly J.R.De Vos, DanielPornillos, OwenDe Vries, Robert P.Porosnicu, MercedesDe Vries, RoryPostigo, AntonioDe Wit, EmmiePoterszman, ArnaudDebbink, KariPrabakaran, MookkanDebnath, Asim KumarPratelli, AnnamariaDebyser, ZegerPrice, PatriciaDeDiego, Marta L.Priyamvada, LalitaDelang, LeenPurdy, MikeDelgado, RafaelPutonti, CatherineDelpeyroux, FrancisPyke, Alyssa T.DeLuca, Neal A.Qi, YipingDelwart, EricQimron, UdiDeng, XufangQin, ZhiqiangDennehy, JohnQiu, Hua-JiDennis, JonathanQiu, JianmingDepledge, Daniel P.Quackenbush, SandraDesdevises, YvesQuer, JosepDesprès, PhilippeRadlinska, MonikaDesselberger, UlrichRagupathy, ViswanathDeStefano, JefferyRai, DevendraDevadas, KrishnakumarRaidal, Shane R.Dhanasekaran, VijaykrishnaRajan, RakhiDi Caro, AntoninoRamirez, ManuelDi Nunzio, FrancescaRamos-Morales, FranciscoDi Serio, FrancescoRandall, Richard EdwardDíaz-Muñoz, Samuel L.Rastelli, EugenioDickson, LauraRavelonandro, MichelDiedrich, SabineRay, KrishanuDiefenbach, Russell J.Redd, AndrewDieterle, María EugeniaReeves, MatthewDietrich, UrsulaRegoes, RolandDietzschold, BernhardReid, St PatrickDivizia, MaurizioReid, StevenDobrovolny, Hana M.Rein, AlanDokland, TerjeReinke, AaronDombrovsky, AvivRemnant, Emily J.Domenech, AnaRenault, TristanDomingo-Calap, PilarRequenna, JesusDreux, MarleneReuter, KlausDrillien, RobertRicciardi, RobertDrulis-Kawa, ZuzannaRichardson, ChristopherDrummer, HeidiRichardson, SimonDu, LanyingRichert-Pöggeler, KatjaDuggal, NishaRichner, JustinDuncan, RoyRigling, DanielDunne, MatthewRima, BertDunowska, MagdalenaRimstad, EspenDutartre, HélèneRinder, MonikaDutch, RebeccaRiska, PaulDyall, JulieRivas, CarmenDykeman, Eric C.Roach, DwayneDziewit, LukaszRobben, JohanEckerle, IsabellaRobek, MichaelEffantin, GrégoryRobert, JacquesEggink, DirkRobinson, Christopher M.Eichwald, CatherineRockman, StevenEiden, Martin E.Rockx, BarryEiras, MarceloRodrigo, IsmaelElbeaino, TouficRodríguez, JavierElder, JohnRodriguez-Andres, JulioEl-Hage, NaziraRodríguez-Carrasco, YelkoElleder, DanielRodriguez-Frias, FranciscoEnderling, HeikoRodriguez-Rubio, LorenaEndersby, Nancy MargaretRohrmann, George FEngeland, Christine E.Roldão, AntónioEriksson, KristinaRomerio, FabioErnst, WolfgangRomero-Brey, InésEschbaumer, MichaelRoques, PierreEsclatine, AudreyRosa, CristinaEssani, KarimRosario, KarynaEstes, Mary K.Rosenthal, KenÉtard, Jean FrançoisRoss, SusanEtebari, KayvanRossetto, Cyprian C.Etienne, LucieRoth, ClaudeEvans, Franklin D.Rousset, DominiqueEwan, PlantRouth, AndrewEwans, MatthewRoux, SimonEyre, NicholasRovnak, JoelFavia, GuidoRowley, Paul AndrewFehr, TonyRoy, ChadFernández, LucíaRückert, ClaudiaFerreira, FernandoRudd, PennyFilipp, DominikRunstadler, JonathanFiorito, FilomenaRussell, RodFischer, MatthiasRyabchikova, Elena I.Fischer, Matthias GRyabov, EugeneFischer, NicoleRyu, SangryeolFish, EleanorSaad, JamilFisher, ChrisSabanadzovic, SaedFisk, John D.Sadaoka, TomohikoFiume, GiuseppeSadiq, S. KashifFletcher, Nicola F.Sadler, AnthonyFlores, RicardoSaelens, XavierFlorin, LuiseSaffarian, SaveezFogle, JonathanSafronetz, DavidFolimonova, SvetlanaSahoo, Malaya KumarFonseca, FilomenaSaitoh, AkihikoFonseca, KevinSaiz, Juan-CarlosFontana, JuanSáiz, MargaritaFox, AdrianSalaheen, SerajusFrada, MiguelSalmons, BrianFradet-Turcotte, AmélieSalvato, MariaFraile, LorenzoSalvatore, MirellaFrancis, Ashwanth ChristopherSalvetti, AnnaFrascaroli, GiadaSanfaçon, HélèneFreiberg, Alexander N.Sano, TeruoFrentiu, FrancescaSantander, JavierFrick, DavidSantiago, Mario L.Fridmann-Sirkis, YaelSantos, Antonio I.Friesen, Paul D.Santos, SílvioFroissart, RémySão-José, CarlosFrossard, Jean-PierreSaptz, StephenFroude, Jeffrey W.Sardo, LucaFuchs, MarcSarmiento, LuisFuji, Shin-ichiSashital, DipaFujisawa, Jun-ichiSatheshkumar, Panayampalli SubbianFujiwara, NaotoSauka, DiegoFukuhara, ToshiyukiSauter, DanielFukushi, HidetoSautto, Giuseppe A.Funnell, SimonSavenkov, EugeneGadea, GillesSavilahti, HarriGaglia, Marta MariaSawa, HirofumiGahlmann, AndreasSawada, ToshikiGaj, ThomasScagnolari, CarolinaGaleazzi, RobertaSchatz, DaniellaGallagher, ThomasSchepens, BertGallay, PhilippeSchildgen, OliverGammon, DonSchmaljohn, AlanGanges, LlilianneSchmidt, RebeccaGanusov, VitalySchmidtke, MichaelaGao, FengSchmitt, AnthonyGaraigorta, UrtziSchols, DominiqueGarcía, MaricarmenScholte, FlorineGarcía, PedroSchubert, UlrichGarcia-Arenal, FernandoSchuetz, AlexandraGarcia-Doval, CarmelaSchuh, AmyGarcia-Ruiz, HernanSchultz-Cherry, StaceyGarfinkel, DavidSchulz, FrederikGarg, RavendraSchur, FlorianGariglio, MarisaSchuurman, Henk-JanGarmann, Rees F.Sciortino, Maria TeresaGarofalo, MariangelaScott, Evan AlexanderGasparrini, MassimilianoSeley-Radtke, KatherineGavioli, RiccardoSen, AdrishGeballe, AdamSeo, Keun-SeokGeller, RonSerra-Moreno, RuthGeoghegan, JemmaServa, SauliusGeorgel, PhilippeSerwer, PhilipGerriets, ValerieSewald, XaverGershon, Anne A.Sgarbanti, MarcoGessain, AntoineShai, YechielGewurz, Benjamin E.Shan, ChaoGhiasi, HomayonShariat, NikkiGhosh, SouvikSheldon, JulieGiam, Chou-Zen (Joe)Shen, WeiranGiansanti, FrancescoShen, YangGifford, RobertSherer, NathanGill, JasonShestopalov, Alexander MGillet, NicolasShi, XiaohongGiotis, Efstathios S.Shi, ZhengliGlasa, MiroslavShih, Wen-LingGo, Yun YoungShil, NirajGoffard, AnneShim, HyunboGomez-Gutierrez, JorgeShimura, HanakoGong, PengShin, Ok SarahGonzalez, GabrielShirafuji, HiroakiGonzález, María EugeniaShirk, PaulGonzalez-Parra, GilbertoShisler, JoannaGonzalez-Reiche, Ana SilviaShoji, IkuoGoodier, Martin R.Short, StevenGoodman, Alan G.Silva, CarlaGóra - Sochacka, AnnaSilva, Laurie A.Gorski, AndrzejSimon, AnneGoupil, Brad ASimulundu, EdgarGourse, RichardSironen, TarjaGowda, MaruthiSitbon, MarcGowen, BrianSkinner, MikeGozmanova, MariyanaSkuja, SandraGrabherr, ReingardSlagle, BettyGralinski, LisaSloan, ChantelGranberg, FredrikSmartt, ChelseaGrand, Roger J.Smith, AmberGrandvaux, NathalieSmith, InaGras, SallySmith, Jason G.Grässer, FriedrichSmith, JessicaGrayfer, LeonSmith, RichardGreen, Timothy J.Smyth, RedmondGreene, CatherineSnyder, ChristopherGregg, RandalSohn, SeonghyangGreiner, TimoSokoloski, KevinGriffiths, AnthonySola, IsabelGrimm, DirkSoloveva, VeronicaGrimsley, NigelSolovyev, AndreyGrose, Julianne HSominskaya, IrinaGroseth, AllisonSong, Min-SukGu, WeifengSong, Yoon-JaeGubala, AnetaSousa, Sílvia A.Gubler, Duane J.Sparger, Ellen (Liz)Guedj, JeremieSpearman, PaulGuillon, ChristopheSpeck, Peter G.Guo, Ju-TaoSpencer, JulietGuo, KejunSpendier, KathrinGuo, Zong ShengSpengler, JessicaHadidi, AhmedSpiegel, MartinHahm, BumsukSpindler, KatherineHall-Mendelin, SonjaSt John, Ashley LHammerl, Jens AndreStahelin, RobertHammond, RosemarieStamminger, ThomasHan, QifengStanton, RichardHaqshenas, GholamrezaStanway, GlynHarder, TimmStapleford, Kenneth A.Hardies, Stephen C.Steger, GerhardHarmon, Karen M.Steger, GertrudHarper, David R.Stehle, ThiloHarrach, BalázsSteinbach, FalkoHarris, JenniferSteinmann, EikeHarrison, RobertStenzel, TomaszHartigan-O’Connor, DennisStephens, EdwardHartman, AmyStevens, ColeHartmann, RuneStevenson, PhilipHasiów-Jaroszewska, BeataStone, DanielHassan, Sherif T. S.Strang, BlairHatoum-Aslan, AsmaStrebel, KlausHattaf, KhalidStrong, JamesHauber, JoachimStrong, JimHayasaka, DaisukeSuárez, Pilar GarcíaHayes, Clair NelsonSullivan, ChrisHayes, Sidney J.Sultana, HameedaHead, StevenSun, CaijunHefferon, Kathleen LauraSun, XiaomingHeidari, MuhammadSupattapone, SurachaiHeinlein, ManfredSusi, PetriHelbig, KarlaSuter, AndyHelle, FrancoisSutter, GerdHendrix, JelleSutton, TroyHepojoki, JussiSutton, Troy CHerker, EvaSuzuki, NobuhiroHernalsteens, Jean-PierreSuzuki, TohruHernandez-Vargas, EstebanSvenningsen, Sine LoHernandez-Vargas, Esteban A.Sweeney, TrevorHerod, Morgan R.Świątek, ŁukaszHersperger, Adam R.Tabernero, DavidHertwig, StefanTai, WanboHewson, IanTakaoka, AkinoriHick, PaulTakashita, EmiHickman, Heather D.Takatsuka, JunHildt, EberhardTakimoto, ToruHinton, DeborahTakizawa, NaokiHirsch, Alec J.Tamba, MarcoHirsch, IvanTamori, AkihiroHiscott, JohnTan, Gene S.Hitchman, RichardTang, QiyiHo, Eric S.Tanji, YasunoriHobson-Peters, JodyTao, PanHoeben, RobTarlinton, RachaelHoffmann, BerndTate, MichelleHoffmann, DonataTatsuya, KatoHohn, ThomasTauriainen, SiskoHolbrook, MichaelTaylor, MatthewHoleva, MariaTaylor, NicholasHolloway, StevenTe Velthuis, AartjanHolmes, EdwardTedbury, PhilipHolmfeldt, KarinTempera, ItaloHoma, Fred L.Temple, LouiseHong, NiTeng, MichaelHorner, StacyTerrier, OlivierHorton, NancyTestoni, BarbaraHorton, Nancy C.Thali, MarcusHorvat, BrankaTheilmann, DavidHosie, MargaretTheriault, StevenHsu, Chih-hungThiel, VolkerHu, GuokuThomas, JulieHu, WenhuiThomason, LynnHuang, Yan-JangThompson, Sunnie R.Hughes, JosephThor, SharmiHuhtamo, EiliThoulouze, Marie IsabelleHulgan, ToddTietjen, IanHummel, MaryTijssen, PeterHusnjak, KoraljkaTincati, CamillaHutchinson, EdwardTohma, KentaroHutt-Fletcher, Lindsey M.Tomaru, YujiHyde, JenniferTonelli, MicheleIkegami, TetsuroTonetti, Michela G.Iorio, Ronald M.Toniolo, Antonio Q.Iranzo, JaimeTorrado, EgídioIsaacs, StuartTorres-Barceló, ClaraIsakova-Sivak, IrinaTortorella, DomenicoIshibashi, KazuhiroToth, ZsoltIshikawa, MasayukiTowner, JonathanIshikawa, TomohiroTran, HienIto, YujiTregoning, John S.Ivanov, Alexander VTriezenberg, Steven J.Iwano, HidetomoTrilling, MirkoIzopet, JacquesTrivedi, PankajJaberolansar, NoushinTrobaugh, DerekJackson, RyanTroyer, RyanJackson, WilliamTruve, ErkkiJacob, GopasTsang, Chi ManJain, PrashantTsukiyama-Kohara, KyokoJain, VaibhavTuomivirta, TeroJakse, JernejTuplin, AndrewJalasvuori, MattiTurina, MassimoJames, DelanoTurnbull, Matthew W.Jan, Fuh-JyhUeda, KeijiJancovich, James KUlaeto, DavidJangra, RohitUldrick, ThomasJanssen, DirkUnger, HermannJavid, NadeemUpton, ChrisJeannot, EmilienUrbanowicz, RichardJehle, JohannesUsui, TatsufumiJiang, ShiboValente, Susana T.Jiménez De Oya, NereidaValle, MikelJochmans, DirkValli, Adrian AlejandroJohnson, NicholasVan Den Wollenberg, Diana J. M.Johnson, Reed F.Van Der Hoek, LiaJończyk-Matysiak, EwaVan Der Kuyl, AntoinetteJones, ClintonVan Doorslaer, KoenraadJones, JeremyVan Engelenburg, SchuylerJones, MelissaVan Etten, JamesJosé Trinidad, Ascencio-IbáñezVan Geelen, AlbertJose, JoyceVan Hemert, MartijnJoshi, AnjaliVan Marle, GuidoJulander, Justin G.van Raaij, MarcJurak, IgorVan Rij, RonaldKageyama, DaisukeVan Santen, VickyKaido, Masanorivan Sinderen, DouweKainov, Denis EVanet, AnneKaján, Győző L.Vaney, Marie-ChristineKalantidis, KritonVanlandingham, DanaKalatzis, Panos G.Varallyay, EvaKalejta, RobertVarani, LucaKaminski, RafalVarjak, MargusKamitani, WataruVarsani, ArvindKanda, TeruVasudevan, Ananda Ayyappan JaguvaKaneko, JunVeit, MichaelKang, Sung HwanVeldhuizen, EdwinKanthikeel, SudheeshVenier, PaolaKantor, BorisVenkatesh, BalanKarganova, Galina G.Ventoso, IvanKarlsson, ErikVenturini, CarolaKároly, FátyolVerheije, HélèneKaruppannan, Anbu KumarVerrier, Eloi RKasamatsu, HarumiVidalakis, GeorgiosKashiwagi, AkikoViejo-Borbolla, AbelKatz, RichardVilibić-Čavlek, TatjanaKaufer, BenediktVimalanathan, SelvaraniKauffman, Kathryn MarieVlasova, Anastasia N.Kaushik, Dr. AjeetVolarevic, VladislavKawaguchi, ToshikazuVon Laer, DorotheeKawakami, JunjiWada, RyuichiKe, RuianWagemans, JeroenKehn-Hall, KyleneWahl-Jensen, VictoriaKelly, DavidWall, DanielKelvin, AlysonWallace, NicholasKennedy, PeterWan, HenryKennedy, Richard B.Wan, YonghongKenney, Scott P.Wang, BillKessels, HelmutWang, Ching-HoKhaperskyy, Denys A.Wang, Chin-TienKielian, MargaretWang, ChunlingKiljunen, SaijaWang, DanKillip, Marian J.Wang, DavidKim, ChonsaengWang, Fun-InKim, MeehyeinWang, HuaiminKim, PeterWang, JenniferKim, Shin-HeeWang, JuexinKim, YunjeongWang, LeyiKimble, SteveWang, QiuhongKinchington, PaulWang, Robert Y.-L.Kindrachuk, JasonWang, YingKinoti, CliffWang, Yun-XingKirui, JamesWang, ZhongdeKlassen, RolandWare, VassieKlimkait, ThomasWarrilow, DavidKlupp, BarbaraWeaver, EricKnierim, DennisWebb, Kimberly M.Knodel, Markus M.Weger, StefanKnust, BarbaraWęgrzyn, AlicjaKobiler, OrenWegrzyn, GrzegorzKoch, GuusWeir, DawnKoloniuk, IgorWeiss, SusanKomatsu, KenWemheuer, FranziskaKonan, KouacouWernery, UlrichKondo, HidekiWeynberg, Karen D.Kong, WeiliWhite, ElizabethKoo, Bon-KyoungWhite, K. AndrewKopecky, JanWhite, RobKoromyslova, AnnaWhitehurst, ChristopherKoshiba, TakumiWhitney, James B.Kotta-Loizou, IolyWhitt, MichaelKrammer, FlorianWichgers Schreur, Paul J.Krapp, ChristianWickner, Reed BKrause, PhilipWiels, JoëlleKremer, EricWight, Darren J.Krenz, BjornWikel, StephenKrenz, BjörnWilkes, Rebecca PenroseKreth, JensWille, HolgerKretschmer, DorotheeWilliams, GrahamKroon, Erna GeessienWilliams, SusanKropinski, AndrewWilliams, TrevorKrug, LaurieWilson, AngusKuang, YangWilson, JoyceKuhar, UrškaWilson, WilliamKuhn, JensWilusz, JeffKukimoto, IwaoWinston, AlanKulakov, LeonidWobus, ChristianeKumar, AshokWolz, ChristianeKumar, BinodWong, Sek ManKumar, MukeshWong, Sek-ManKümmerer, Beate M.Wu, Hung-YiKunz, StefanWu, ZhiweiKurosu, TakeshiWylie, KristineKurz, Jacqueline LaRoseWylie, Stephen J.Kwak, Jong HwanXia, NingshaoKwang, HeoXia, YuchenKyei, George BXiang, Shi-huaLa Scola, BernardXiao, Chuan (River)Laanto, ElinaXing, JunjiLakatos, LorantXu, WenxingLakdawala, SeemaYaegashi, HajimeLamba, DorianoYamashita, MakotoLamp, BenjaminYang, Hung-ChihLan, KeYang, ZhiLongLang, AndrewYasuike, MotoshigeLanglois, RyanYau, ShereeLangridge, WilliamYeam, InhwaLau, Susanna K. P.Young, Howard A.Lauber, ChrisYount, Jacob S.Lauring, AdamYuen, Kwok-YungLavazza, AntonioYukl, Erik T.Lavezzo, EnricoYurochko, AndrewLawrence, MartinZago, MiriamLeclerc, DenisZanini, FabioLecointe, FrançoisZell, RolandLee, Moo-SeungZhang, HuanminLee, Seung-JooZhang, QiongLefeuvre, PierreZhang, QiyaLegrand-Abravanel, FlorenceZhang, RongLehman, SusanZhang, RuiLeitao, AlexandreZhang, YijunLemay, GuyZheng, Yong-HuiLens García, SabelaZhou, DongmingLeroux, CarolineZhou, GraceLeroy, BaptisteZhou, Heshan SamLesbarres, DavidZhou, JieLever, Andrew M. L.Zhou, ShuntaiLevy, LauraZhu, JingenLeyrat, CedricZhu, LuchangLi, DapengZhu, QiyunLi, FengZidovec Lepej, SnjezanaLi, GuoQingZiegelbauer, JosephLi, Mei-LingZimmer, GertLi, MelodyZimmermann, CosimaLi, ShitaoZmora, PawelLi, YanZoll, JanLi, YizeZoltán, ZádoriLiang, ChenZschach, HenrikeLibeau, GenevièveZúñiga, SoniaLicciardello, Grazia


